# Machine learning-based stratification of Parkinson’s disease progression using dysautonomia symptoms and transcriptomic signatures

**DOI:** 10.1016/j.gendis.2025.101831

**Published:** 2025-08-22

**Authors:** Su-Jin Baek, Hyeong Joon Jun, Jae Hyeok Han, SeoHyun Lee, HuiYan Zhao, Muhammad Umar, Jae Young Jang, Jung-Hee Jang

**Affiliations:** a Korean Medicine Data Division, Korea Institute of Oriental Medicine, Daejeon 34054, Republic of Korea; b School of Electrical, Electronics, and Communication Engineering, Korea University of Technology and Education, Cheonan 31253, Republic of Korea; c Insight Mining Co., Ltd., 151 Noeun-ro, Yuseong-gu, Daejeon 34087, Republic of Korea; d Korean Medicine Science Research Division, Korea Institute of Oriental Medicine, 1672 Yuseongdae-ro, Yuseong-gu, Daejeon 34054, Republic of Korea; e Korean Convergence Medical Science, University of Science & Technology, School of Korea Institute of Oriental Medicine, Daejeon 34054, Republic of Korea

Although autonomic dysfunction (AutD) is a prognostic factor for Parkinson’s disease (PD), little is known regarding AutD progression-based stratification and its association with transcriptomic signatures. We examined the association between gene expression levels and risk factors according to the disease progression rate based on longitudinal changes of AutD severity in individual patients with PD. In total, 612 patients with PD with available SCales for Outcomes in Parkinson’s disease-autonomic (SCOPA-AUT) data from the Parkinson Progression Marker Initiative (PPMI) were included. A personalized hidden Markov model (HMM) was used to define the disease STATEs based on SCOPA-AUT scores. K-means clustering revealed three clusters reflecting STATE transition, with clusters 1 to 3 indicating rapid progression. Cox proportional hazard models showed that cluster 3 and gastrointestinal tract (GIT) symptoms in AutD significantly increased the risk for moderate stage PD. Furthermore, differential expression analyses revealed cluster-specific genes and molecular mechanisms associated with PD pathogenesis and GIT symptoms. Candidate molecular targets related to GIT symptoms were associated with rapid progression in patients with PD, suggesting that GIT-associated molecular targets could be used to stratify patients with PD showing different progression rates and thus facilitate personalized treatment strategies.

PD is clinically heterogeneous, where some patients experience a relatively benign course while others rapidly progress to disability. AutD in PD patients is a prognostic factor serving as an independent determinant of rapid disease progression and short survival. It is considered a non-motor marker for classifying different PD subtypes.^1^ Considering the importance of expression profiling in disease for understanding the pathological mechanisms and for predicting onset, progression, and treatment effectiveness, we used large-scale longitudinal data from the PPMI study to determine whether expression signatures and risk factors are prognostically associated with disease progression rates over time from PD onset based on SCOPA-AUT (Table S1). The detailed methods are shown in Data S1. The data used to prepare this article were openly available from the PPMI database (https://www.ppmi-info.org/accessdata-specimens/download-data) and were downloaded on June 22, 2023. For pre-processing, 21 SCOPA-AUT features from the PPMI database were decreased to 10 new features using principal component analysis, and 612 patients with PD with 10 extracted features were included. Details of the ten newly extracted principal components are illustrated in Figure S1. The patient characteristics are listed in Table S2 and a flow chart of the overall study design is shown in Figure S2. To stratify the STATEs of AutD severity across the 612 participants, we used the HMM and classified eight STATEs in the SCOPA-AUT time series. The average scores and severity levels of individual AutD items across the eight STATEs are shown in Tables S3 and S4. Progression from STATEs 1 to 8 generally indicated increasing severity of PD clinical symptoms (Table S5). Notably, STATE 8 had significantly fewer participants than the other STATEs (Table S6). K-means clustering was used to classify clusters based on STATE level transmission over time for each patient (Table S7); the number of clusters (K) was tested from 2 to 10, and the optimal number (*K* = 3) was determined based on the steepest part observed in the Scree plot (Fig. S3). We mainly selected three clusters considering the STATE-level shift patterns: STATEs 2 to 3 in Cluster 1, STATEs 3 to 5 in Cluster 2, and STATEs 5 to 7 in Cluster 3 (Table S8). The severity of clusters 1 to 3 was generally indexed as rapid progression (Fig. S4 and Table S9). In the investigation of risk factors for reaching terminal disease severity, cluster 3 and the GIT domain were more associated with an increased risk of reaching the terminal stage, as indicated by the MDS-UPDRS score 57 in cluster 3 (HR, 2.21; 95% CI, 1.50–3.26; *P* < 0.00), the GIT domain (HR, 2.39; 95% CI, 1.66–3.43; *P* < 0.00), and the STATE terminal 7 level in cluster 3 (HR, 147.55; 95% CI, 20.51–1061.40; *P* < 0.00) and in the GIT domain (HR, 3.45; 95% CI, 2.28–5.21; *P* < 0.00) (Fig. 1A–D and Table S10). We further analyzed the hazard ratio of individual symptoms within the GIT domain (Table S11 and Fig. S5), and items including sialorrhea or constipation and straining for defecation were associated with an increased risk of disease exacerbation. Additionally, a network analysis to investigate high-centrality items among the GIT domains for the three clusters (Table S12) revealed that constipation and defecation straining appeared frequently across clusters 1, 2, and 3.

**Figure 1:**
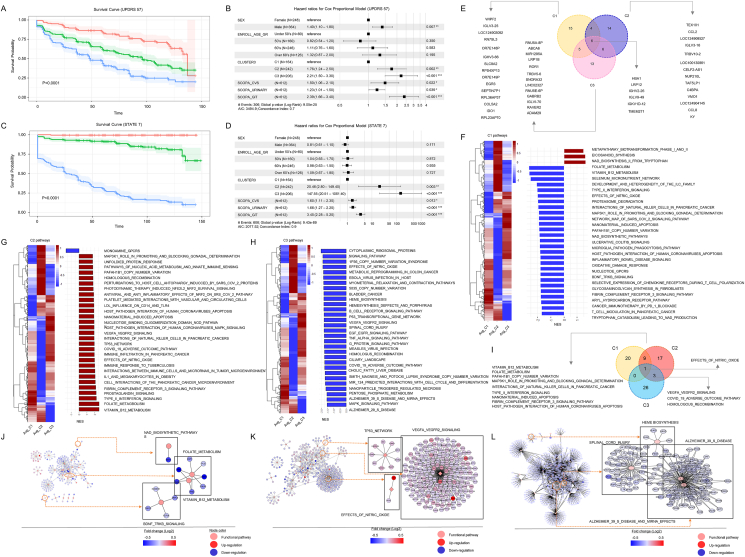
Identification of gene expression signatures and risk factors according to disease progression patterns based on longitudinal AutD severity in patients with Parkinson’s disease. Kaplan–Meier survival curves of the cumulative probability **(A, C)** and hazard ratio for autonomic dysfunction symptoms **(B, D)** based on the severity of the MDS-UPDRS score (A and B) and terminal STATE 7 level (C and D). In Fig. 1A and C, the red, green, and blue curves represent Clusters 1, 2, and 3, respectively. Gene expression signatures by three clusters of progressive patterns. Venn diagram showing the number of genes and cluster genes across the top 30 cluster DEGs **(E)**. Identification of enriched molecular pathways in the three clusters. Heatmap and bar plot showing the DEGs and enriched pathways of Cluster 1 **(F)**, Cluster 2 **(G)**, and Cluster 3 **(H)**. The heatmap represents cluster-DEGs, and the blue/red color depicts down/up-regulation in cluster groups compared to non-cluster groups. The bar plot represents cluster-enriched molecular pathways, and the blue/red bar depicts negative/positive values of the normalized enrichment score of the cluster. Venn diagram showing the number of enriched pathways in the three clusters. Identification of functional networks in the three clusters **(I)**. Functional network of Cluster 1- **(J)**, Cluster 2- **(K)**, and Cluster 3-enriched pathways **(L)**.

To identify the gene expression signatures for each cluster, we performed DEG analysis of the three clusters. The top 30 DEGs in each cluster (Fig. S6 and Table S13) and the Venn diagram comparing the DEGs across the three clusters (Fig. 1E, Fig. S7, and Table S14) were obtained. In cluster 1, early growth response 3 (*EGR3*) (logFC: −0.515 and *P* = 1.49 × 10^−9^), one of 21 genes^2^ differentiating ulcerative colitis and Crohn’s disease from normal samples, and indoleamine 2,3-dioxygenase 1 (*IDO1*) (logFC: −0.369 and *P* = 1.51 × 10^−8^), which is positively correlated with irritable bowel syndrome severity,^3^ were down-regulated compared to those in non-cluster 1. These results suggest that *EGR3* and *IDO1* expression are associated with GIT symptoms and have the potential to be biomarkers for predicting early PD. In cluster 3, Ribonucleoprotein, PTB Binding 2 *RAVER2* (logFC = 0.302 and *P* = 7.92 × 10^−9^), which is associated with susceptibility to ulcerative colitis,^4^ and Gamma-Aminobutyric Acid Type A Receptor Subunit Beta2 (*GABRB2*) (logFC: 0.335 and *P* = 2.08 × 10^−9^) were up-regulated compared to those in non-cluster 3. Gene set enrichment analysis targeting Wiki pathways was performed to identify the major molecular mechanisms for each cluster. The top 30 enriched molecular pathways and a heatmap of the DEGs in each cluster are shown in Figure 1F–H and Table S15, and the enriched pathways are compared across the clusters in Figure 1I. The functional network-enriched pathways and square boxes for each cluster are shown in Figure 1J–L and Figure S8–S10, respectively. In cluster 1, enriched molecular pathways, including neuroprotective factors such as “BDNF TRKB signaling” (NES = −1.72, *P* = 0.0287), “folate metabolism” (NES = −2.69, *P* = 0.0021), “NAD biosynthetic pathways” (NES = −1.87, *P* = 0.0158), and “Vitamin B12 metabolism” (NES = −2.62, *P* = 0.002), were down-regulated (Fig. 1J). Most cluster 2 molecular pathways were up-regulated compared to those in non-Cluster2. In Cluster 2, oxidative stress regulators, including “VEGFA VEGFR2 signaling” (NES = 1.93 and *P* = 0.0077), “TP53 network” (NES = 1.94 and *P* = 0.0035), and “effects of nitric oxide” (NES = 2 and *P* = 0.0018), were up-regulated (Fig. 1K). In cluster 3, the top 30 enriched molecular pathways were significantly down-regulated. Among these, “HEME biosynthesis,” “Spinal cord injury,” “Alzheimer disease (AD), and the miRNA effect,” were more significantly down-regulated (Fig. 1L). Notably, “spinal cord injury,” associated with an increased risk of PD and GIT-related diseases during the 3-year follow-up,^5^ was significantly detected in cluster 3, with the highest severity. Overall, transcriptome signature analysis is useful for predicting the severity of PD symptoms as well as the target genes that regulate them.

These findings underscore the importance of individual AutD symptoms and gene expression profiles in predicting PD progression. Future research is needed to validate these biomarkers and explore therapeutic interventions targeting GIT symptoms to ultimately develop personalized treatment strategies and improve outcomes for patients with PD.

## CRediT authorship contribution statement

**Su-Jin Baek:** Formal analysis, Writing – original draft, Conceptualization, Methodology. **Hyeong Joon Jun:** Formal analysis, Writing – original draft. **Jae Hyeok Han:** Methodology, Formal analysis. **SeoHyun Lee:** Methodology. **HuiYan Zhao:** Formal analysis. **Muhammad Umar:** Formal analysis. **Jae Young Jang:** Writing – original draft, Methodology, Writing – review & editing, Conceptualization. **Jung-Hee Jang:** Writing – original draft, Supervision, Writing – review & editing, Conceptualization.

## Ethics approval

Ethical approval for data analysis was obtained from the Institutional Review Board of the Korea Institute of Oriental Medicine (I-2312/012-004).

## Funding

This research was supported by grants from the 10.13039/501100003725National Research Foundation of Korea funded by the Korean government (NRF-2021R1I1A2048890); the Korea Health Technology R&D Project through the Korea Health Industry Development Institute (KHIDI); the Ministry of Health & Welfare, Republic of Korea (RS-2025-02222724); and the Education and Research Promotion Program of KOREATECH in 2025. The Parkinson Progression Markers Initiative (PPMI), a public-private partnership, is funded by the Michael J. Fox Foundation for Parkinson’s Research and its funding partners (www.ppmiinfo.org/about-ppmi/who-we-are/study-sponsors).

## Conflict of interests

The authors declare no conflict of interests.
