# Supplementary material for: Machine learning-based stratification of Parkinson’s disease progression using dysautonomia symptoms and transcriptomic signatures
Source: Genes Dis. 2025 Aug 22;13(3):101831. doi: 10.1016/j.gendis.2025.101831 (PMC12854859; doi:10.1016/j.gendis.2025.101831)
Supplement: Multimedia component 1 [file mmc1.docx]

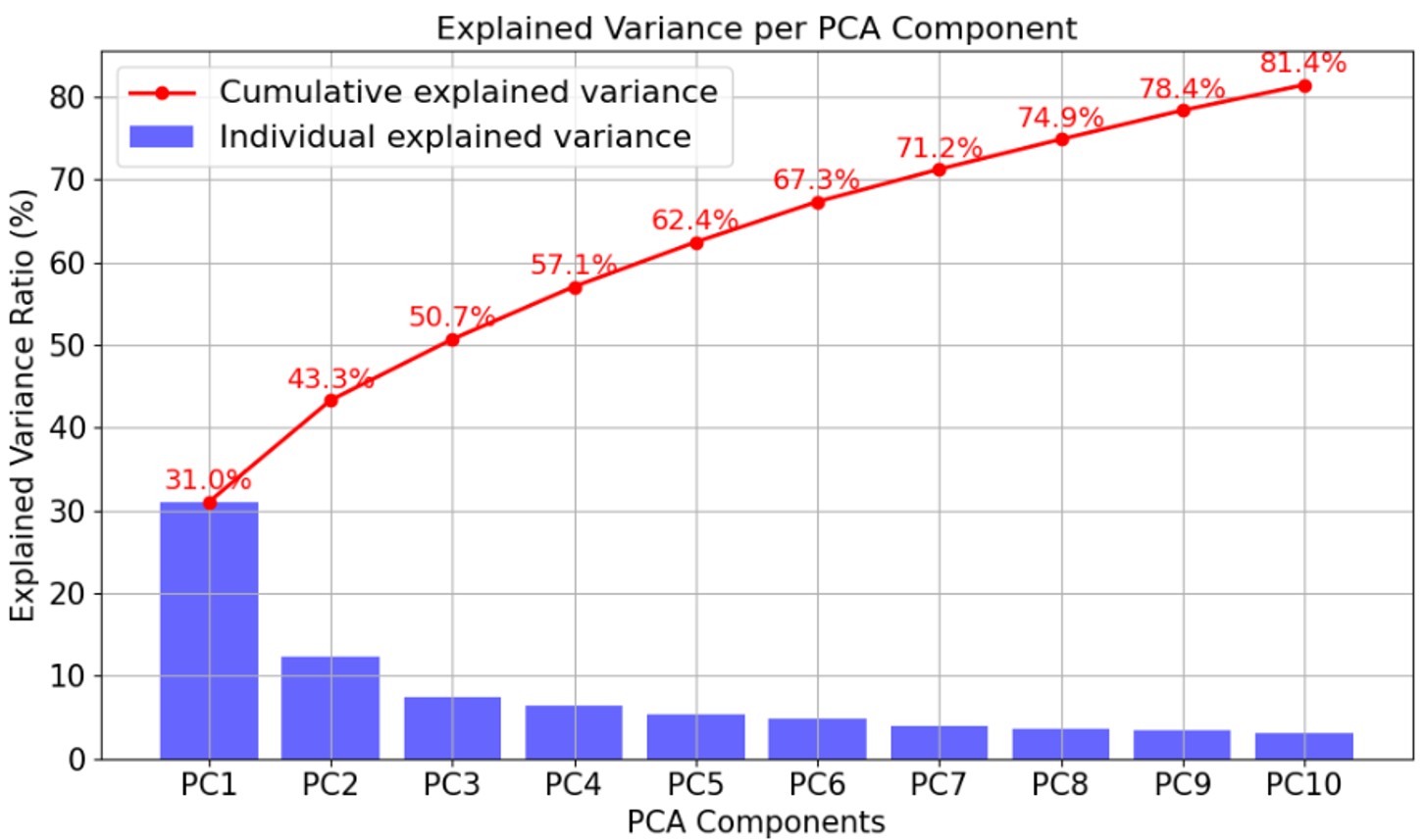


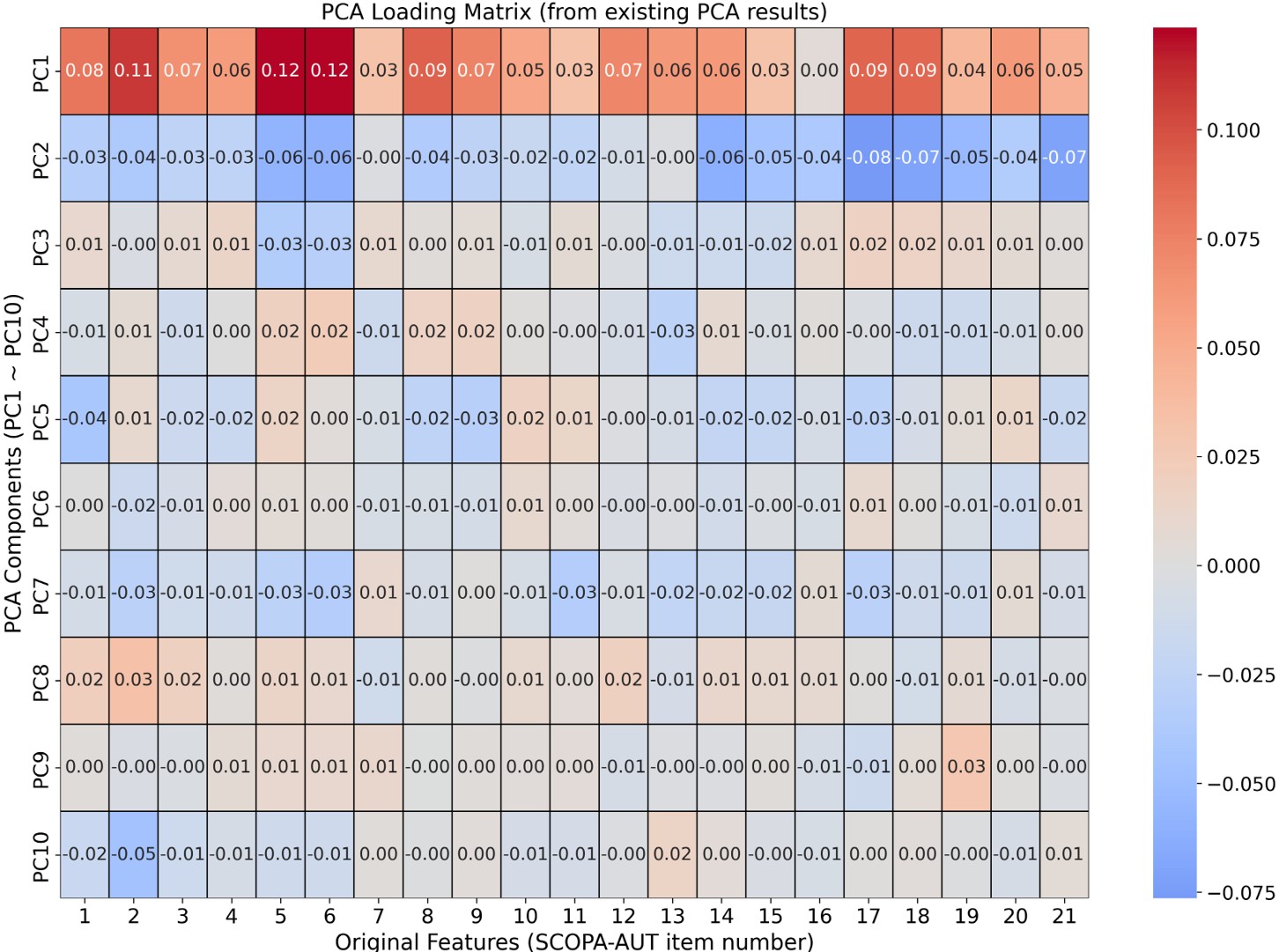


Figure S1. The ten new features derived from principal component analysis (PCA). (A) Scree plot showing the individual and cumulative explained variance ratios of the ten principal components derived from PCA. (B) Heat-map of the PCA loading matrix showing the relationship between the original SCOPA-AUT features and the ten principal components (PC1–PC10).


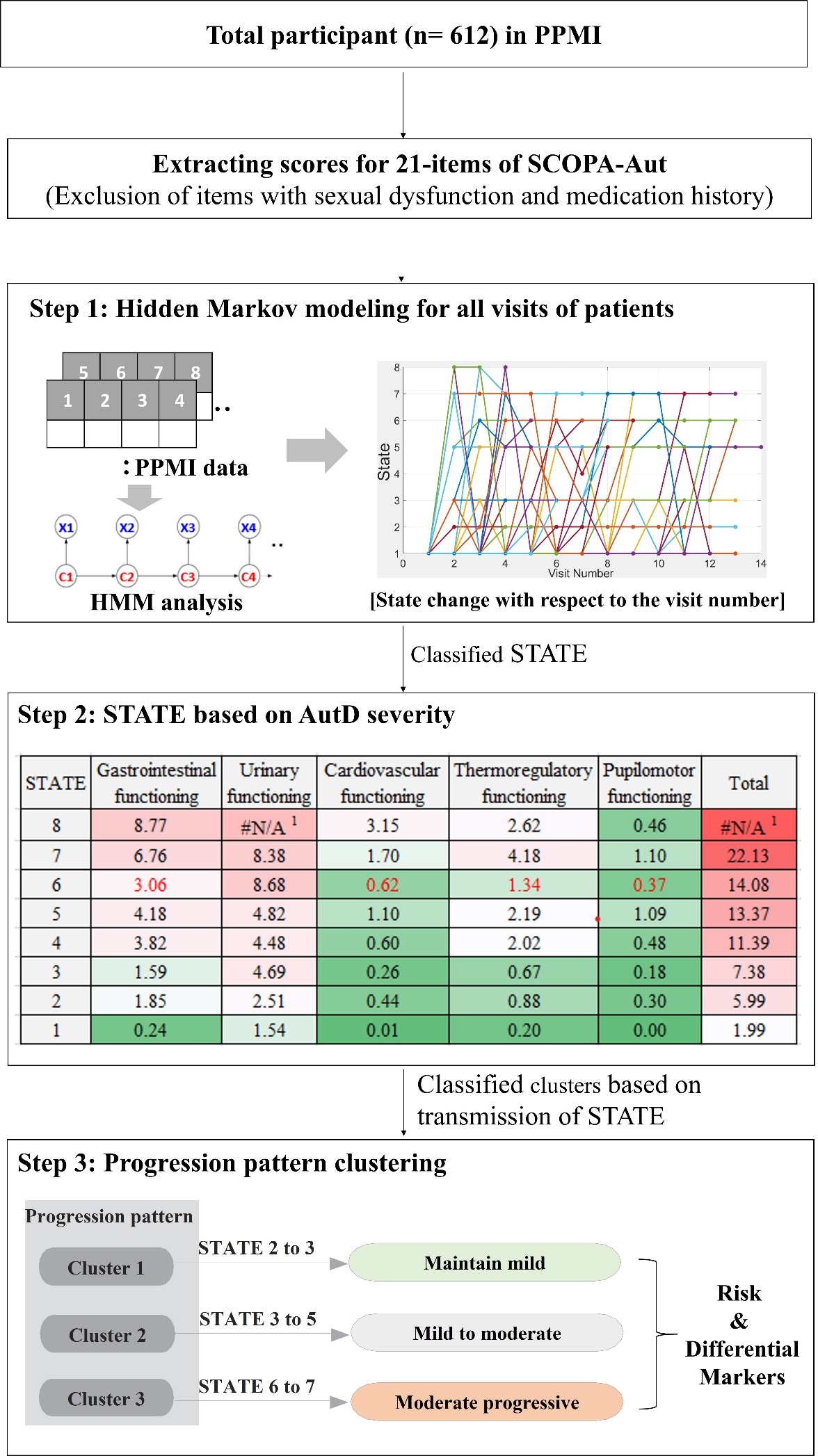


Figure S2. Study design flowchart showing details of the data analysis. The open-source hmmlearn library in Python (https://pypi.org/project/hmmlearn/) was used to apply the HMM to analyze the time-series data. After creating a model with ‘viterbi’ decode algorithm, the model was trained using the 10 extracted features data of 612 patients. Through this process, the changes in each patient’s STATE over time can be derived. K-means clustering method to group patients based on the transmission of STATE-levels over time for analysis.


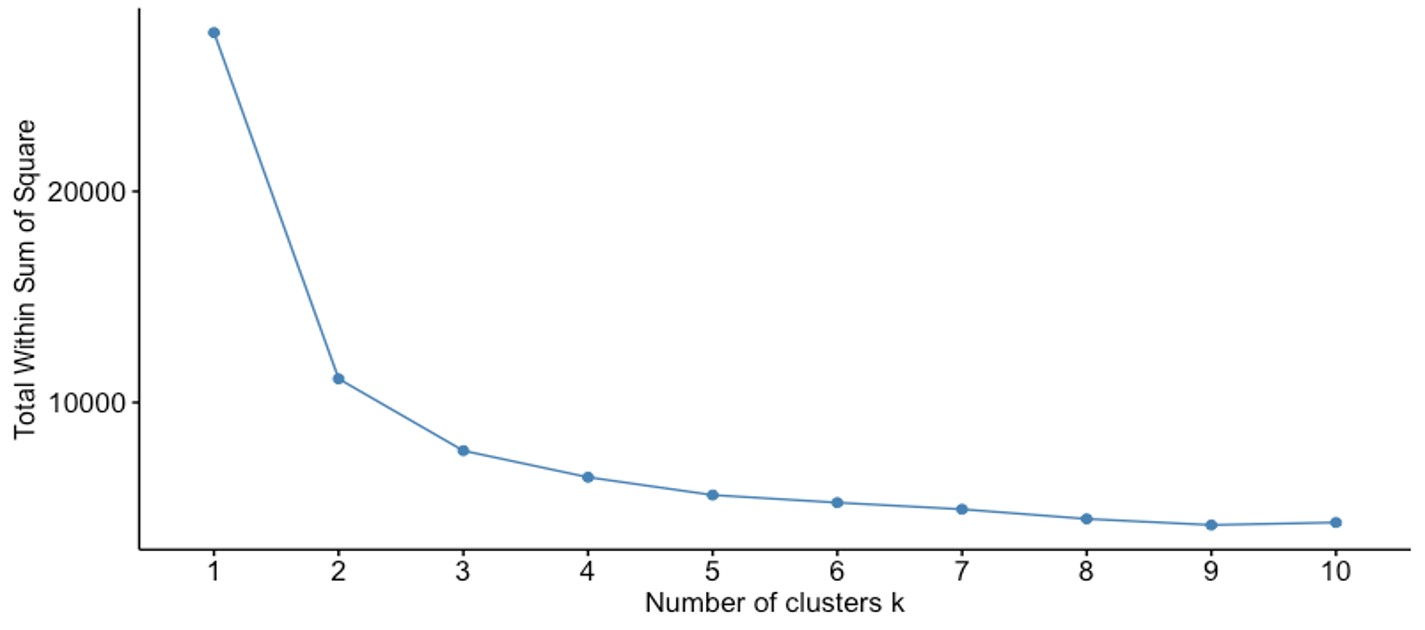


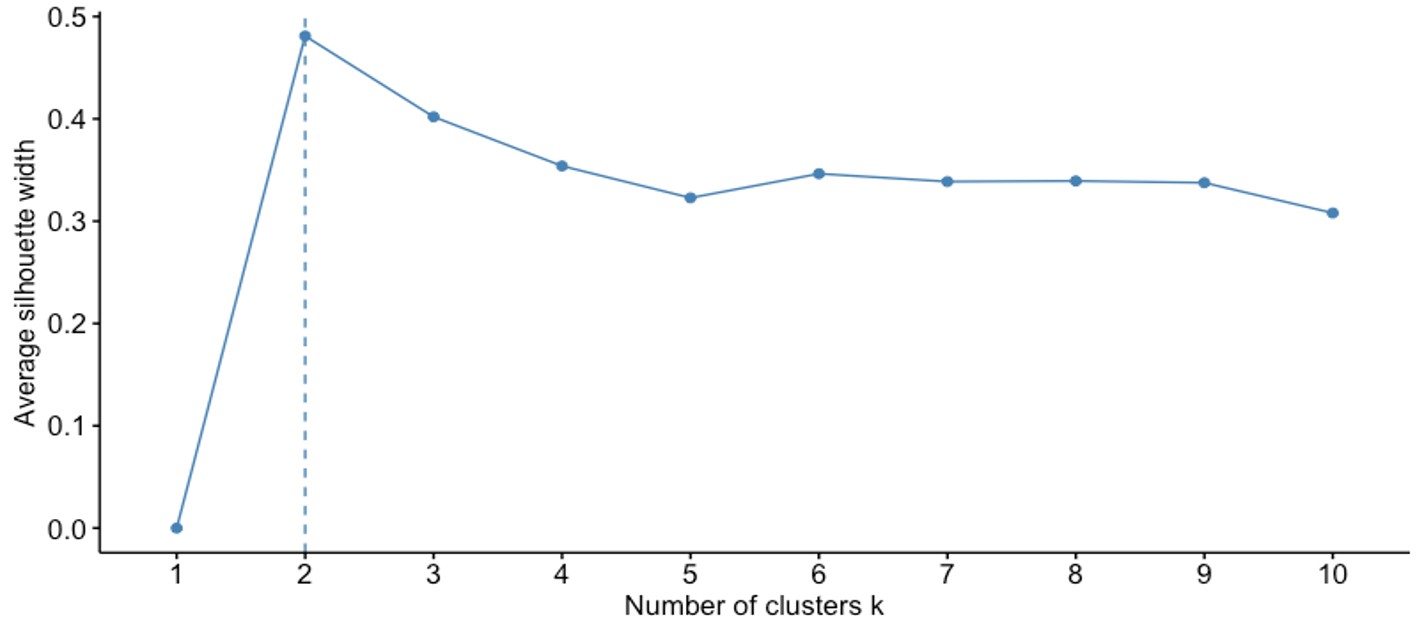


Figure S3. Scree plot for selection of optimal K number using elbow and silhouette method. The number of cluster K is shown on the X-axis and characteristic values on the Y-axis. Each node represents a STATE, which is ranged according to its characteristic value


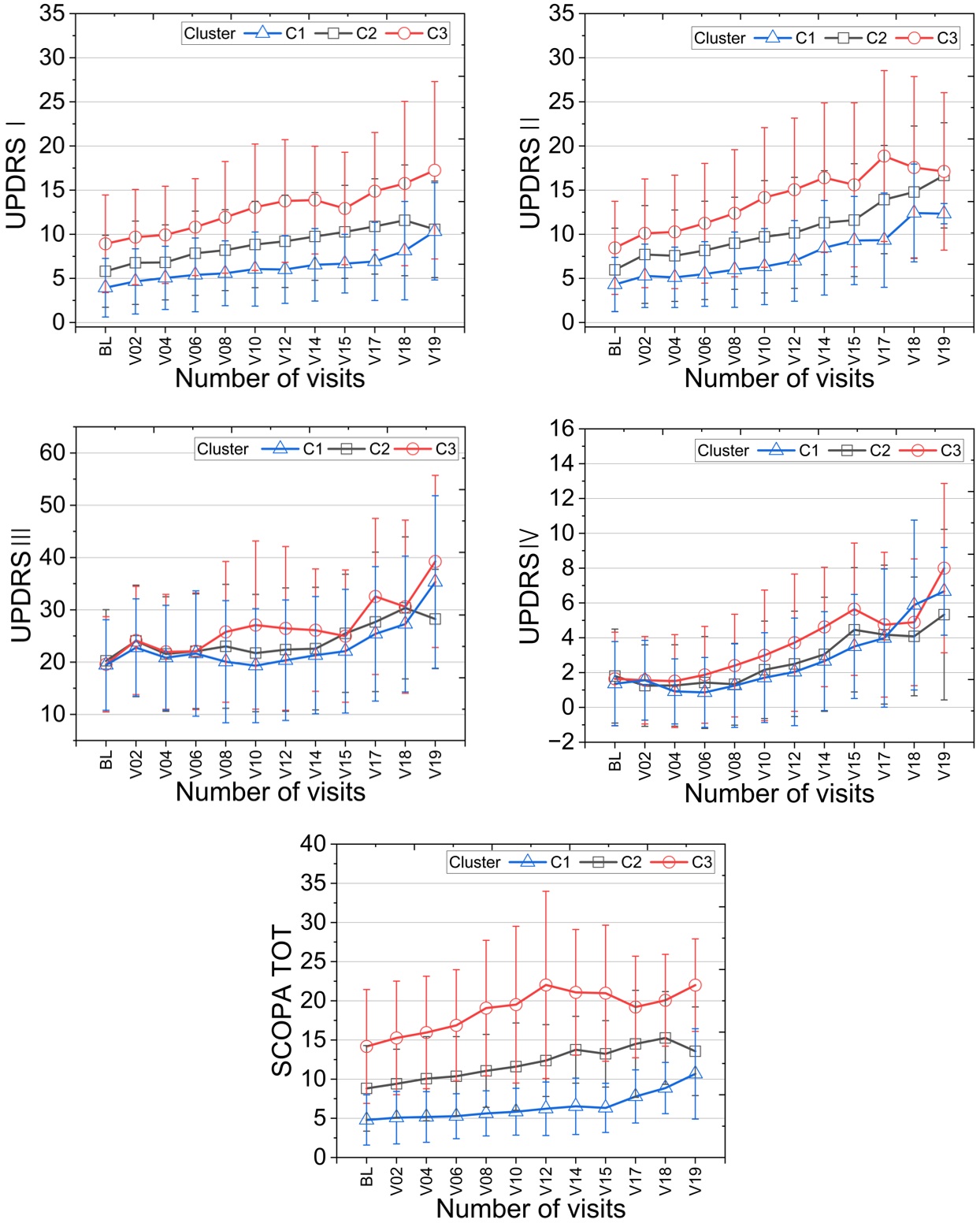


Figure S4. Changes in SCOPA-Aut and MDS-UPDRS by cluster according to visit schedule. The number of visits is shown on the X-axis and score values on the Y-axis


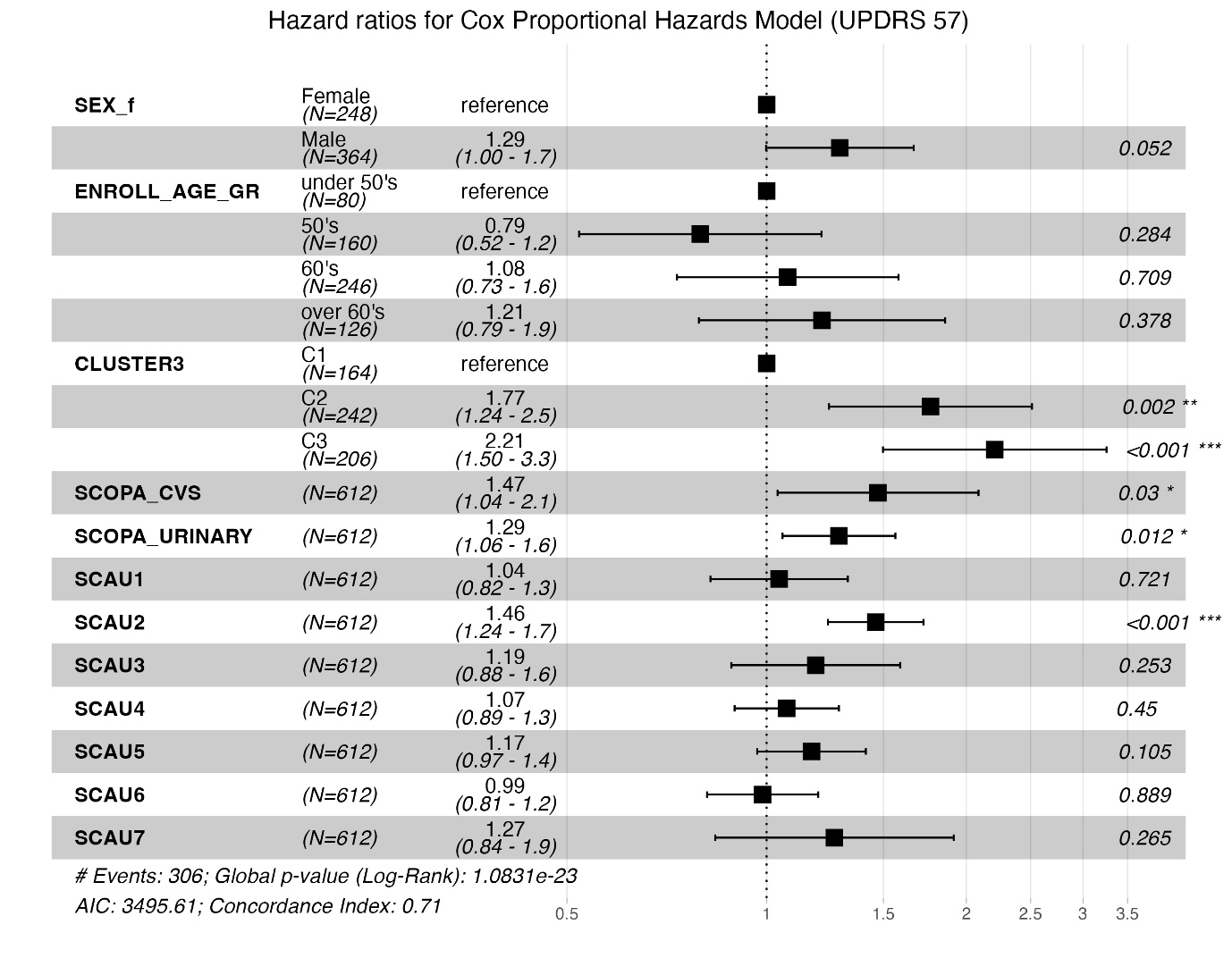


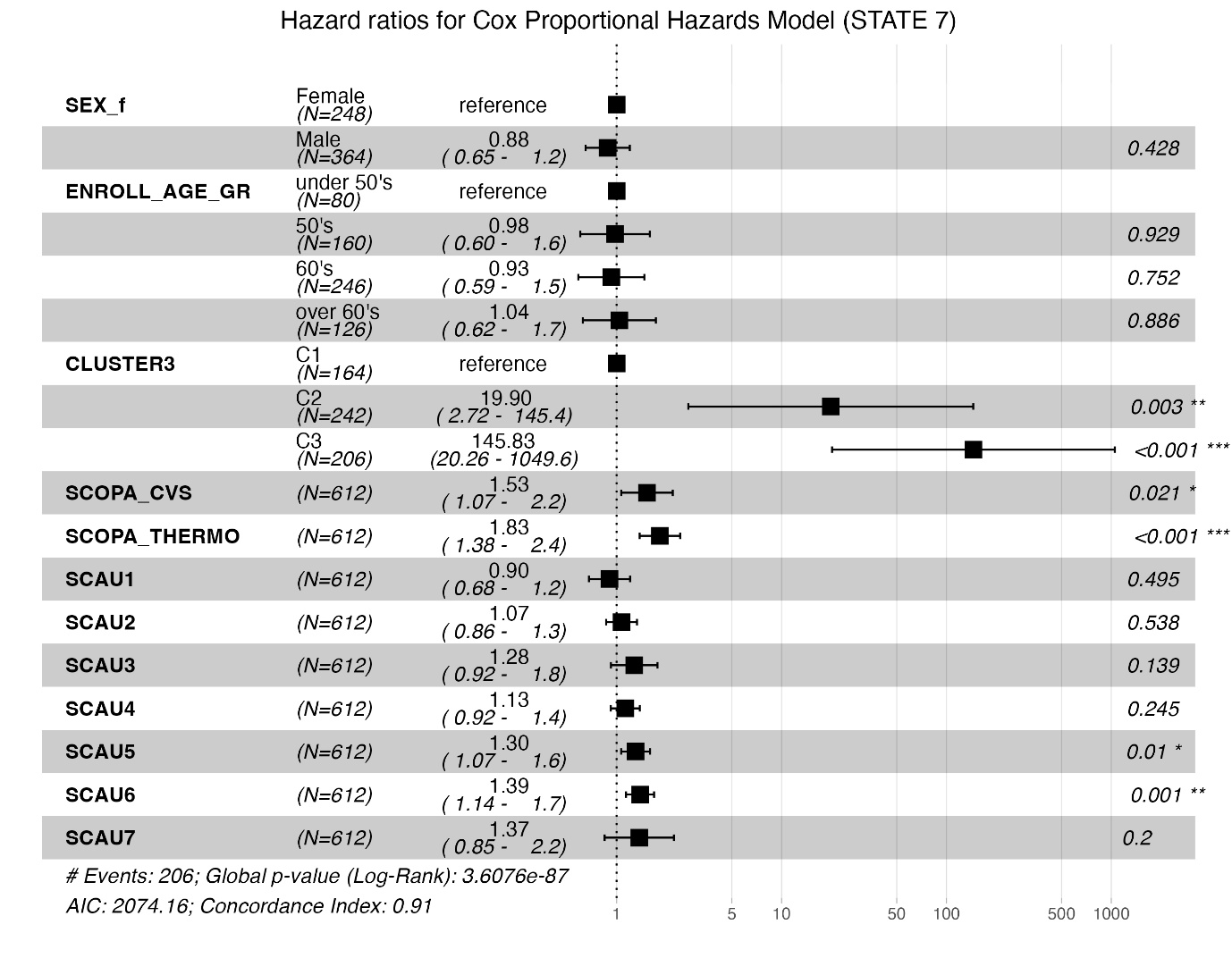


Figure S5. Cox Proportional Hazards Regression Models of Individual Autonomic Symptoms of GIT domain to reach moderate stage of Sever UPDRA score (A) and terminal STATE 7 level (B)


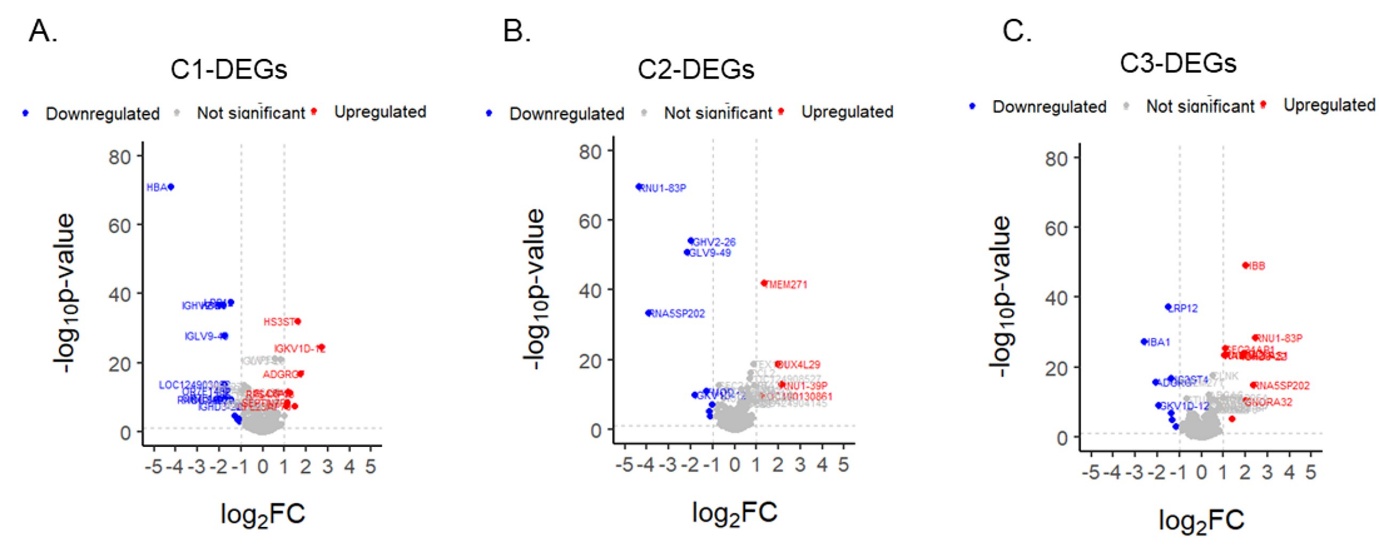


Figure S6. Gene expression signatures by three clusters of progressive patterns. Volcano plot of top 30 DEGs for Cluster 1- (A), Cluster 2-, (B), and Cluster 3- (C) DEG groups. Blue/red color represents the down/up-regulated genes in cluster group compared to the non-cluster group.


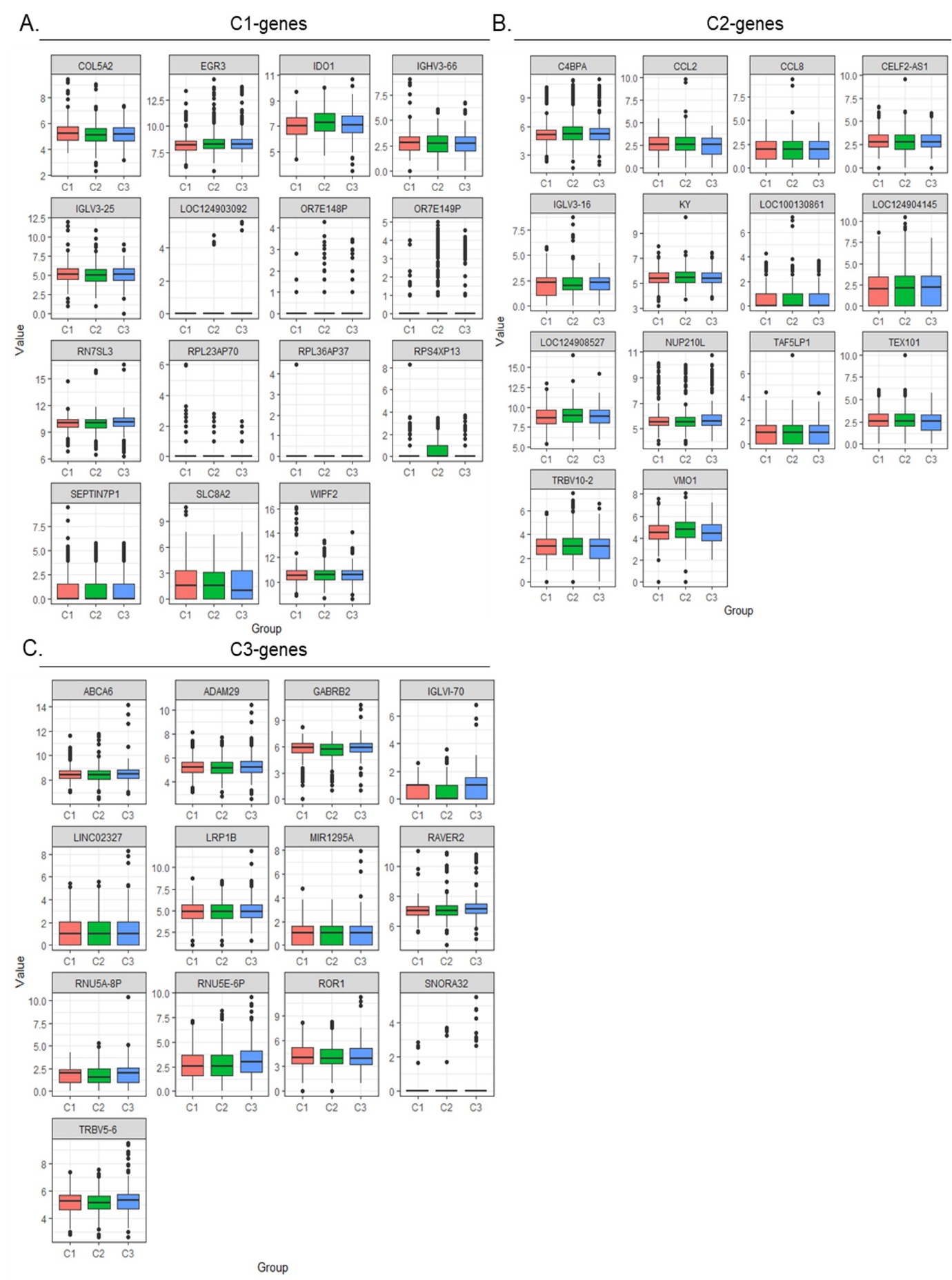


Figure S7. Comparison of gene expression values in cluster specific genes. Box plot represents gene expression values of (A) Cluster 1-, (B) Cluster 2-, and (C) Cluster 3-genes.


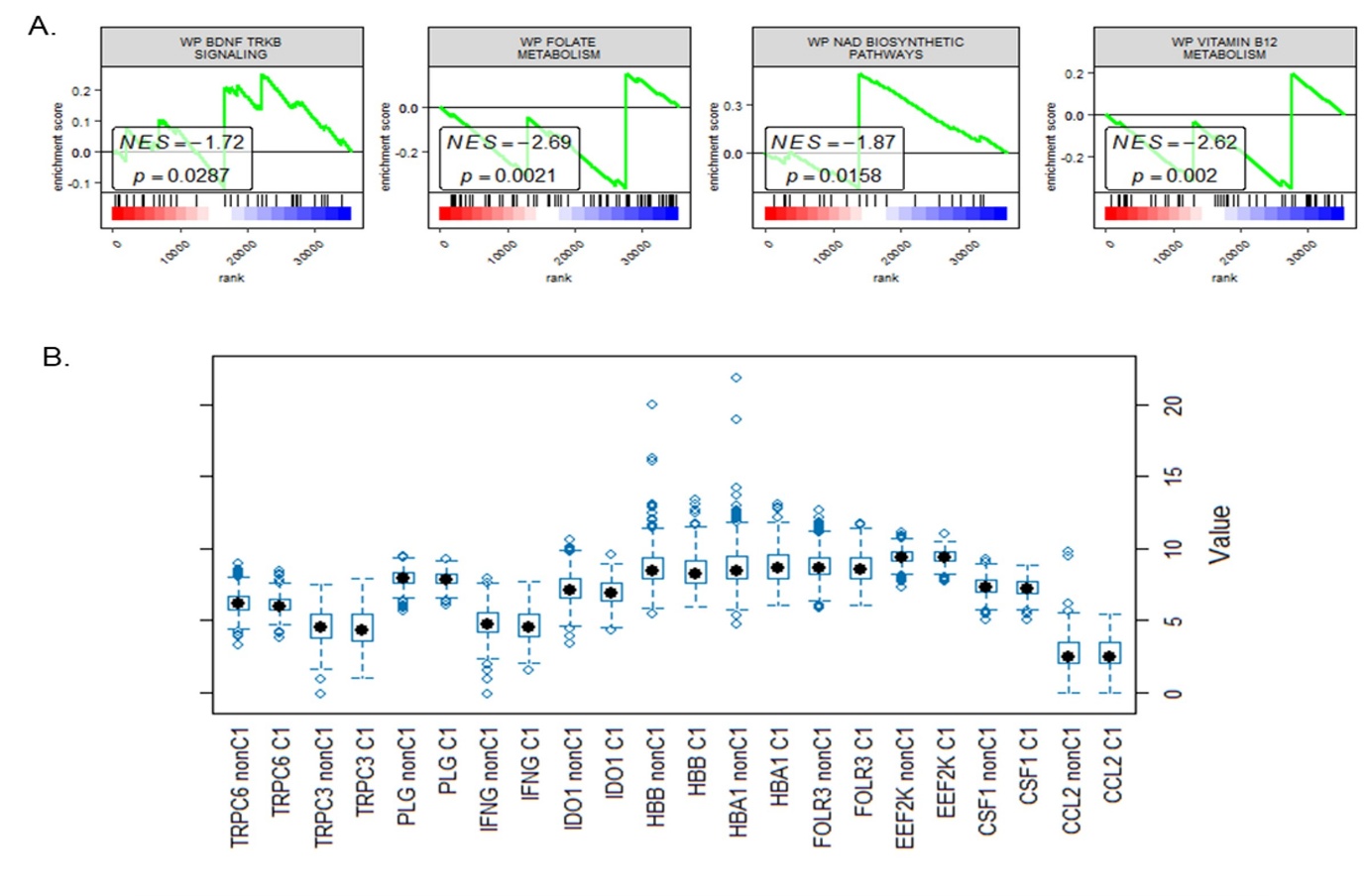


Figure S8. Enriched pathway and core genes of Cluster 1-specific group. (A) GSEA plot for BDNF TRKB signaling, folate metabolism, NAD biosynthetic pathways, and vitamin B12 metabolism. (B) Box plot for core genes of functional network in Cluster 1-specific group.


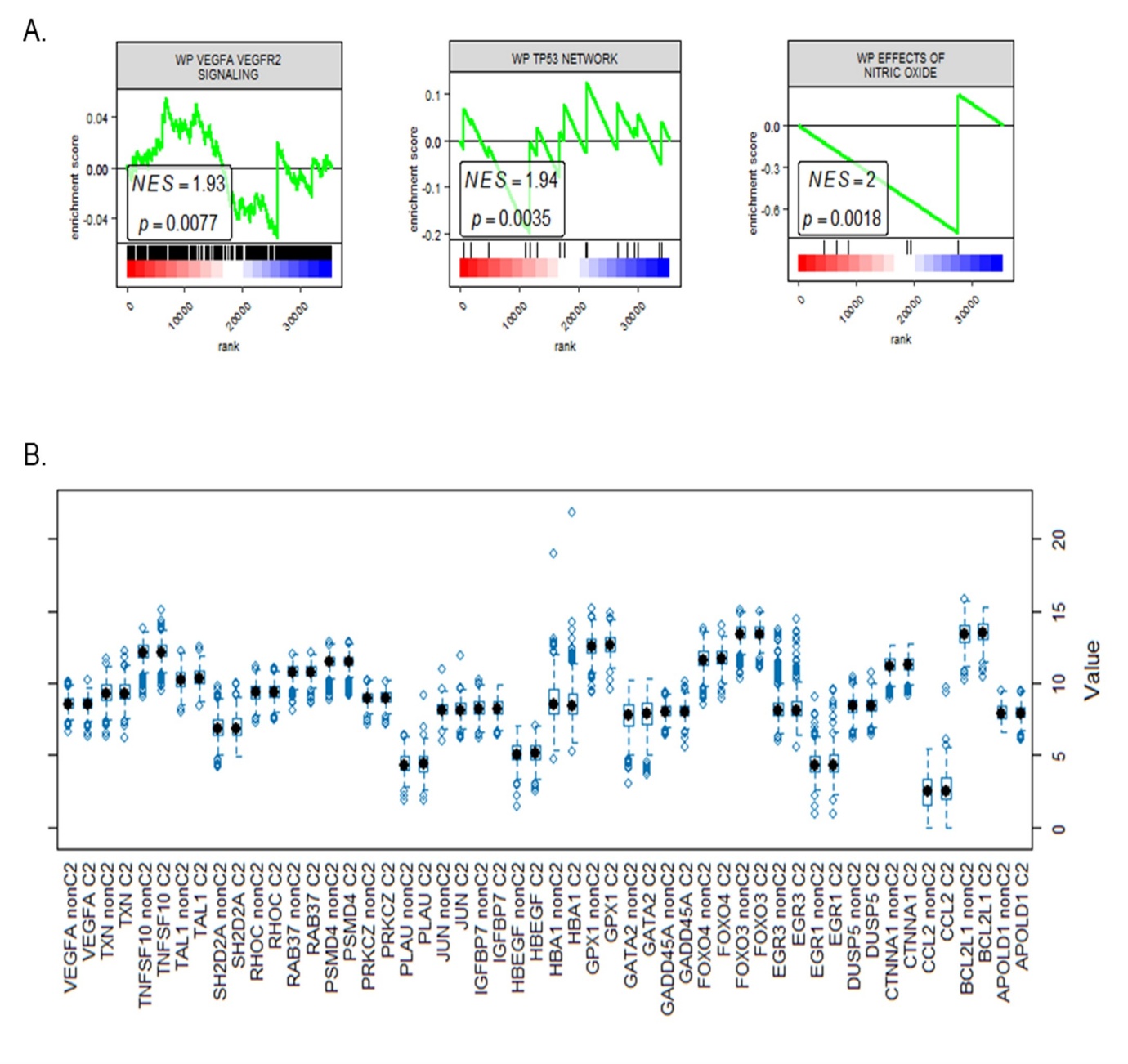


Figure S9. Enriched pathway and core genes of Cluster 2-specific group. (A) GSEA plot for VEGFA-VEGFR2 signaling, TP53 network, and effects of nitric oxide pathway. (B) Box plot for core genes of functional network in Cluster 2-specific group.


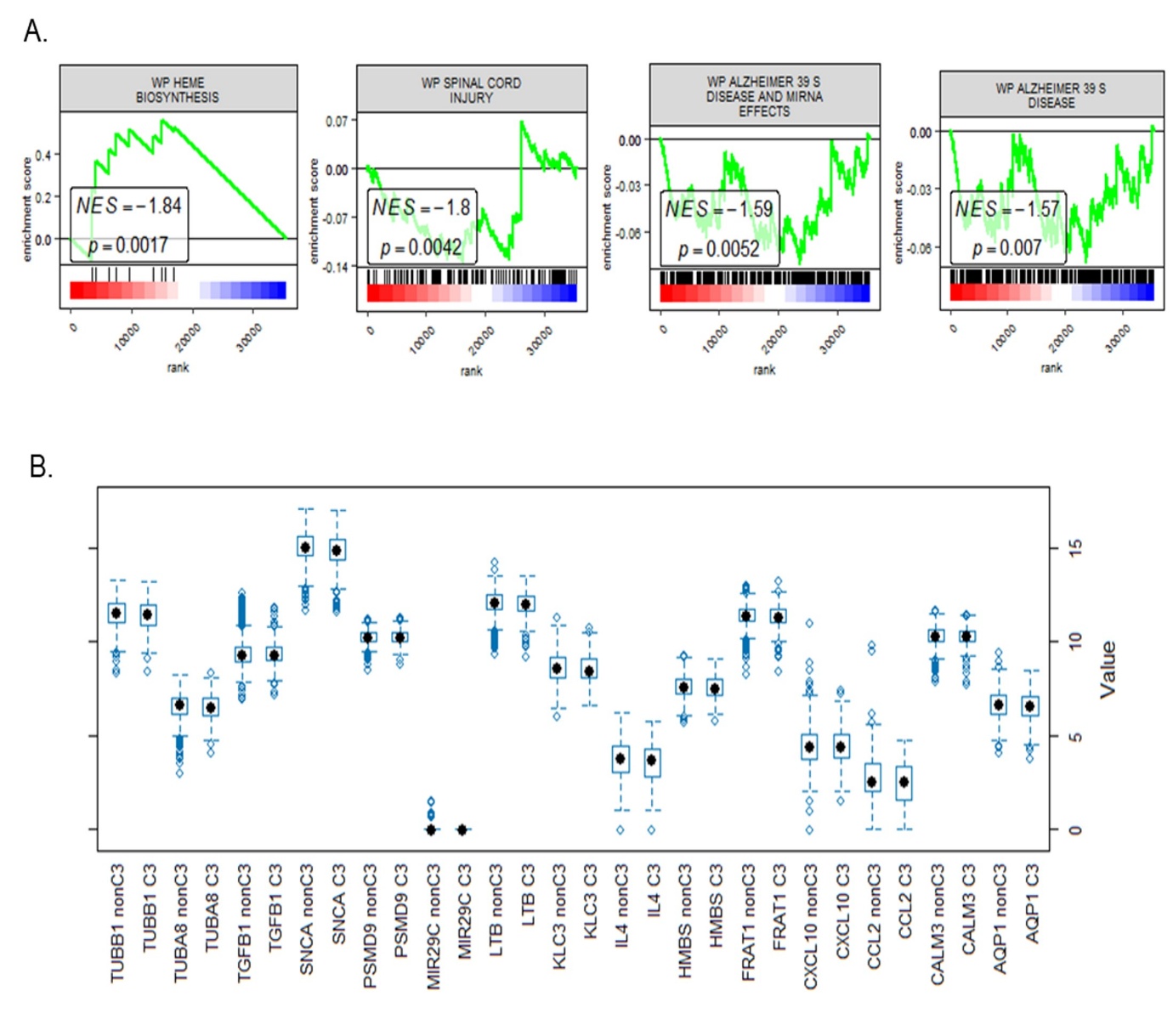


Figure S10. Enriched pathway and core genes of Cluster 3-specific group. (A) GSEA plot for HEME biosynthesis, Spinal cord injury, Alzheimer disease and miRNA effects, and alzheimer disease. (B) Box plot for core genes of functional network in Cluster 3-specific group.
