# Supplementary material for: Machine learning-based stratification of Parkinson’s disease progression using dysautonomia symptoms and transcriptomic signatures
Source: Genes Dis. 2025 Aug 22;13(3):101831. doi: 10.1016/j.gendis.2025.101831 (PMC12854859; doi:10.1016/j.gendis.2025.101831)
Supplement: Multimedia component 2 [file mmc2.docx]

**Supplementary data 1. Detailed Methods**

**Study designs and participants**

This prospective, international multicenter longitudinal data from the PPMI to identify biomarkers for PD progression. Detailed methods are outlined in subsequent sections and Figure 1. Detailed methodology for the PPMI study is available on their website. The data used to prepare this article were openly available from the PPMI database (https://www.ppmi-info.org/accessdata-specimens/download-data). For updated information on this study, see [www.ppmi-info.org](http://www.ppmi-info.org).

The inclusion criteria All PD participants are as follows. ^1-3^

a) Male or female age 30 years or older at Screening Visit.

b) Has a clinical diagnosis of Parkinson’s disease at Screening Visit.

c) Patients must have at least two of the following: resting tremor, bradykinesia, rigidity (must have either resting tremor or bradykinesia); OR either asymmetric resting tremor or asymmetric bradykinesia.

d) Individuals taking any of the following drugs: alpha methyldopa, methylphenidate, amphetamine derivatives or modafinil, must be willing and medically able to hold the medication for at least 5 half-lives before SPECT imaging.

e) Able to provide informed consent.

f) Confirmation that participant is eligible based on centrally determined criteria for the University of Pennsylvania Smell Identification Test (UPSIT) completed through other recruitment efforts.

g) Either is male, or is female and meets additional criteria below, as applicable: Female of childbearing potential who is not pregnant, lactating, or planning pregnancy during the study and has a negative pregnancy test on day of Baseline SPECT imaging test prior to injection of DaTscanTM.

The exclusion criteria All PD participants are as follows.

a) Atypical PD syndromes due to either drugs (e.g., metoclopramide, flunarizine, neuroleptics) or metabolic disorders (e.g., Wilson’s disease), encephalitis, or degenerative diseases (e.g., progressive supranuclear palsy).

b) A clinical diagnosis of dementia as determined by the investigator.

c) Previously obtained MRI scan with evidence of clinically significant neurological disorder (in the opinion of the Investigator).

d) Received any of the following drugs: dopamine receptor blockers (neuroleptics), metoclopramide and reserpine within 6 months of Screening visit.

e) Current treatment with anticoagulants (e.g., coumadin, heparin, oral thrombin inhibitors) that might preclude safe completion of the lumbar puncture.

f) Condition that precludes the safe performance of routine lumbar puncture, such as prohibitive lumbar spinal disease, bleeding diathesis, or clinically significant coagulopathy or thrombocytopenia.

g) Any other medical or psychiatric condition or lab abnormality, which in the opinion of the investigator, might preclude participation.

h) Any other reason that, in the opinion of the investigator, would render the participant unsuitable for study enrollment.

The participant eligibility can be found on the PPMI website under “Research Documents and SOPs | Parkinson's Progression Markers Initiative". Inclusion criteria for our analysis required a primary diagnosis of PD, more than four hospital visits and at least two or more study visits with SCales for Outcomes in PArkinson's disease - autonomic (SCOPA-AUT) and Movement Disorder Society–sponsored revision of the Unified Parkinson’s Disease Rating Scale (MDS-UPRDS) assessments. The study was approved by the Institutional Review Board of each site, and all participants provided written informed consent. This study used the Strengthening the Reporting of Observational Studies in Epidemiology (STROBE) reporting guidelines. The data were downloaded on June 22, 2023 from the prospective, observational, longitudinal cohort study utilizing annual visit data from participants registered between July 2010 and June 2023 in the Parkinson Progression Marker Initiative (PPMI). The data were analyzed from June 21, 2023, to June 08, 2024.

**Clinical Assessments**

Clinical assessments included the SCOPA-AUT, MDS-UPDRS, and HY scales. PD symptom severity was assessed using MDS-UPDRS Parts I ~ III, where higher scores indicate worse motor function.^4^ Part Ⅳ was excluded owing to missing data on dopamine drug usage at the baseline.^5^ The SCOPA-AUT, a 25-item questionnaire, evaluated autonomic symptoms (eTable 1 in the Supplement).^6^ For clustering all participants, the evaluation of sexual dysfunction divided by sex was excluded, focusing solely on items 1 to 21 of the SCOPA-AUT. The HY scale includes stages 1 through 5.^3^

**Data pre-processing**

In all, 21 SCOPA-AUT features were decreased to 10 new features using Principal Component Analysis (PCA) for improved longitudinal data accuracy. The selection of 10 new features was based on the criterion that they should together account for more than 80% of the total variance in the original data. This means that we chose the smallest number of principal components (10 in this case) such that their combined contribution to explaining the variability in the data exceeded 0.8 in terms of the cumulative explained variance ratio.^7,8^ The explained variance ratios for each of the ten principal components, as well as their cumulative contributions, are shown in figure S1A. Notably, the first principal component (PC1) alone accounts for 31.0% of the total variance, and the cumulative variance explained by the first three components exceeds 50%. All ten components together explain approximately 81.4% of the variance, supporting the appropriateness of using these components to capture the essential structure of the original feature space while minimizing information loss. To interpret the relationship between the original features and the derived PCs, a loading matrix was constructed. Each entry in this matrix represents the contribution of a raw feature to a given principal component, effectively indicating how strongly the original variables influence the direction of the respective PCs as shown in figure S1B. Among the ten extracted components, PC1 and PC2 explained the largest proportion of variance in the original features, making them the most representative components for interpretation. Based on the loading structure, PC1 was mainly influenced by Features associated with SCOPA-AUT item number2, 5, and 6, which correspond to Gastrointestinal dysfunction characteristics, whereas PC2 was shaped by Features associated with SCOPA-AUT item number 17, 18, and 21, commonly associated with Thermoregulatory dysfunction. This indicates that different principal components may reflect distinct clinical domains. Finally, longitudinal data of 612 patients with PD along with 10 extracted features were obtained and applied to the HMM.

**Modelling**

The study employed an HMM to analyze PD progression over time using the decreased SCOPA-AUT features.^9,10^ HMM defines hidden states in time-series data and probabilistically infers state changes over time. Eight STATEs were identified in the SCOPA-AUT time series of the 612 patients.^11^ The open-source hmmlearn library in Python (<https://pypi.org/project/hmmlearn/>) was used to apply the HMM for analyzing the time-series data.

**Clustering**

We employed the K-means clustering method to group patients based on the transmission of STATE-levels over time for analysis. Clusters were defined based on the observed STATEs during each patient's scheduled visits. In cases where a patient missed a scheduled visit, the final observation was recorded. Three distinct clusters were identified using the scree plot, each exhibiting unique patterns of STATE transitions over time. Because of challenges in assessing the severity of urinary symptoms using a catheter (questions 8–13 of urinary functioning), these symptoms were not scored and are indicated as #N/A. Instead, by analyzing scores from the MDS-UPDRS I (1-10 urinary problems) question, we confirmed that urinary symptoms were the most severe in STATE 8.

**Statistical analysis**

To assess the association between time to two terminal events–STATE 7 (given the small number of patients reaching STATE 8) or an MDS-UPDRS I–III total score of 57 (moderate stage of PD)^12^ –patients were divided into three clusters using K-means. Kaplan–Meier curves were plotted to visualize survival probabilities. To compare survival distributions among the clusters for terminal events, log-rank tests were conducted, followed by Bonferroni post-hoc tests for inter-group comparisons. Associations between time to these events and SCOPA-Aut scores were explored using Cox regression analysis. Covariates included demographic variables (SEX and AGE groups) and cluster ID. Variable selection for SCOPA-Aut scores was conducted using the backward method. Proportional hazard assumptions were met for the STATE 7 event, but for MDS-UPDRS 57, time-dependency required the inclusion of interaction terms. In addition, within the Cox model, significant GIT scores were disaggregated into their seven constituent items for detailed analysis, aiming to identify specific significant contributors within the GIT score.

**Transcriptome analysis**

The counts and transcripts per million (TPM) matrix data of bulk RNA sequencing (RNA-seq) were obtained from the PPMI cohort (baseline, n=330; visit 4, n=243; visit 6, n=262; and visit 8, n=253). Differential expression analysis between groups (non-Cluster1 vs. Cluster1, non-Cluster2 vs. Cluster2, and non-Cluster3 vs. Cluster3) was conducted using the edgeR v.4.0.16 R package.^13^ Gene set enrichment analysis (GSEA) was performed on pre-ranked expression values according to DEG analysis using fgsea (v.1.12.0; <http://bioconductor.org/packages/fgsea/>).^14^ The Wiki pathway gene set, used as the input, was obtained from the Molecular Signature Database (MSigDB).^15^ The top 30 enriched pathways were selected based on their P value (< 0.05). Network analysis of GSEA results was performed using Cytoscape (v.3.10.0).^16^

**Network analysis**

The corresponding SCOPA-Aut items were then formatted as “SCAUX-Y” (where X represents the item number and Y represents the score), and their simple and co-occurrence frequencies were calculated. Cases with a score of zero were excluded. For co-occurrence calculations, we counted the number of pairs across all items (SCAUX-Y) for each participant and summed these pairs across all participant visits to determine the co-occurrence frequency. These frequency data were then used to generate visual networks using Cytoscape software (ver. 3.10.1). Centrality analysis of the network, was also conducted using Cytoscape.

**References**

1. Marek K, Chowdhury S, Siderowf A, et al. The Parkinson's progression markers initiative (PPMI) - establishing a PD biomarker cohort. *Ann Clin Transl Neurol.* 2018;5(12):1460-1477.

2. Parkinson Progression Marker I. The Parkinson Progression Marker Initiative (PPMI). *Prog Neurobiol.* 2011;95(4):629-635.

3. Wang L, Zhang W, Liu F, et al. Association of Cerebrospinal Fluid Neurofilament Heavy Protein Levels With Clinical Progression in Patients With Parkinson Disease. *JAMA Netw Open.* 2022;5(7):e2223821.

4. Goetz CG, Fahn S, Martinez-Martin P, et al. Movement Disorder Society-sponsored revision of the Unified Parkinson's Disease Rating Scale (MDS-UPDRS): Process, format, and clinimetric testing plan. *Mov Disord.* 2007;22(1):41-47.

5. Holden SK, Finseth T, Sillau SH, Berman BD. Progression of MDS-UPDRS Scores Over Five Years in De Novo Parkinson Disease from the Parkinson's Progression Markers Initiative Cohort. *Mov Disord Clin Pract.* 2018;5(1):47-53.

6. Visser M, Marinus J, Stiggelbout AM, Van Hilten JJ. Assessment of autonomic dysfunction in Parkinson's disease: the SCOPA‐AUT. *Movement disorders: official journal of the Movement Disorder Society.* 2004;19(11):1306-1312.

7. Suhr DD. Principal component analysis vs. exploratory factor analysis. *SUGI 30 proceedings.* 2005;203(230):1-11.

8. Bartholomew DJ. Principal Components Analysis. In:2010:374-377.

9. Schuster-Böckler B, Bateman A. An introduction to hidden Markov models. *Curr Protoc Bioinformatics.* 2007;Appendix 3:Appendix 3A.

10. Kwon BC, Anand V, Severson KA, et al. DPVis: Visual Analytics With Hidden Markov Models for Disease Progression Pathways. *IEEE Trans Vis Comput Graph.* 2021;27(9):3685-3700.

11. Severson KA, Chahine LM, Smolensky LA, et al. Discovery of Parkinson's disease states and disease progression modelling: a longitudinal data study using machine learning. *Lancet Digit Health.* 2021;3(9):e555-e564.

12. Martínez-Martín P, Rodríguez-Blázquez C, Mario A, et al. Parkinson's disease severity levels and MDS-Unified Parkinson's Disease Rating Scale. *Parkinsonism Relat Disord.* 2015;21(1):50-54.

13. Robinson MD, McCarthy DJ, Smyth GK. edgeR: a Bioconductor package for differential expression analysis of digital gene expression data. *Bioinformatics.* 2010;26(1):139-140.

14. Subramanian A, Tamayo P, Mootha VK, et al. Gene set enrichment analysis: a knowledge-based approach for interpreting genome-wide expression profiles. *Proc Natl Acad Sci U S A.* 2005;102(43):15545-15550.

15. Liberzon A, Birger C, Thorvaldsdóttir H, Ghandi M, Mesirov JP, Tamayo P. The Molecular Signatures Database (MSigDB) hallmark gene set collection. *Cell Syst.* 2015;1(6):417-425.

16. Shannon P, Markiel A, Ozier O, et al. Cytoscape: a software environment for integrated models of biomolecular interaction networks. *Genome Res.* 2003;13(11):2498-2504.
